# Exploring a multimodal approach for utilizing digital biomarkers for childhood mental health screening

**DOI:** 10.3389/fpsyt.2024.1348319

**Published:** 2024-04-11

**Authors:** Myounglee Choo, Doeun Park, Minseo Cho, Sujin Bae, Jinwoo Kim, Doug Hyun Han

**Affiliations:** ^1^ HCI Lab, Yonsei University, Seoul, Republic of Korea; ^2^ Department of Psychiatry, College of Medicine, Chung-Ang University, Seoul, Republic of Korea

**Keywords:** digital biomarkers, children’s mental health, multimodal digital biomarkers, heart rate variability, eye-tracking, voice

## Abstract

**Background:**

Depression and anxiety are prevalent mental health concerns among children and adolescents. The application of conventional assessment methods, such as survey questionnaires to children, may lead to self-reporting issues. Digital biomarkers provide extensive data, reducing bias in mental health self-reporting, and significantly influence patient screening. Our primary objectives were to accurately assess children’s mental health and to investigate the feasibility of using various digital biomarkers.

**Methods:**

This study included a total of 54 boys and girls aged between 7 to 11 years. Each participant’s mental state was assessed using the Depression, Anxiety, and Stress Scale. Subsequently, the subjects participated in digital biomarker collection tasks. Heart rate variability (HRV) data were collected using a camera sensor. Eye-tracking data were collected through tasks displaying emotion-face stimuli. Voice data were obtained by recording the participants’ voices while they engaged in free speech and description tasks.

**Results:**

Depressive symptoms were positively correlated with low frequency (LF, 0.04–0.15 Hz of HRV) in HRV and negatively associated with eye-tracking variables. Anxiety symptoms had a negative correlation with high frequency (HF, 0.15–0.40 Hz of HRV) in HRV and a positive association with LF/HF. Regarding stress, eye-tracking variables indicated a positive correlation, while pNN50, which represents the proportion of NN50 (the number of pairs of successive R-R intervals differing by more than 50 milliseconds) divided by the total number of NN (R-R) intervals, exhibited a negative association. Variables identified for childhood depression included LF and the total time spent looking at a sad face. Those variables recognized for anxiety were LF/HF, heart rate (HR), and pNN50. For childhood stress, HF, LF, and Jitter showed different correlation patterns between the two grade groups.

**Discussion:**

We examined the potential of multimodal biomarkers in children, identifying features linked to childhood depression, particularly LF and the Sad.TF:time. Anxiety was most effectively explained by HRV features. To explore reasons for non-replication of previous studies, we categorized participants by elementary school grades into lower grades (1st, 2nd, 3rd) and upper grades (4th, 5th, 6th).

**Conclusion:**

This study confirmed the potential use of multimodal digital biomarkers for children’s mental health screening, serving as foundational research.

## 1 Introduction

### 1.1 Childhood depression, anxiety, stress

Depression and anxiety are common mental health issues in children and adolescents. The prevalence of depression and anxiety among children has consistently risen, with 5.6 million children diagnosed with anxiety and 2.4 million with depression in the United States in 2020. These figures represent increases of 27% and 29%, respectively, compared to 2016 (1). The situation in South Korea mirrors this trend. According to the National Health Insurance Service (NHIS), the number of children and adolescents aged 18 and under receiving treatment for depression or anxiety disorders rose from 54,000 in 2019 to 63,000 in 2021, reaching approximately a total of 210,000 from 2019 to 2022 (2). Depression and anxiety often coexist in children (3). Some researchers argue that anxiety and depression in preadolescents are part of a larger structure of negative emotions, rather than being distinct emotional states (4). In adults, the cumulative effect of daily stress plays a significant role in predicting the development of depressive and anxiety symptoms (5, 6). This phenomenon is also observed in children (7).

Beck’s Cognitive Theory of Depression suggests that some individuals possess negative cognitive schemas, and when activated by factors such as stress, these schemas contribute to the emergence of negative perceptions and interpretations, potentially leading to depressive symptoms (8). Research on depression in children and adolescents also supports the Cognitive Vulnerability Theory of Depression (9). Stressors related to school or home environments are associated with higher levels of depressive symptoms and aggression in children (10). Children experiencing depression encounter a higher number of stressful life events, such as an increase in arguments with parents, compared to their non-depressed counterparts (11).

Furthermore, stress is associated with anxiety. Studies examining the relationship between non-traumatic life stress events (e.g., parental divorce, a grandparent’s death, or relocation) and anxiety disorders or symptoms have shown that children diagnosed with anxiety disorders have experienced stressors more frequently than non-anxious children (12). Similarly, families experiencing an increased number of stressors report higher levels of anxiety symptoms in both parents and children (13).

### 1.2 Challenges in screening for the mental health of children

The early detection and evaluation of mental health issues in children are crucial for implementing prevention and intervention strategies (14). Mental health concerns in children under the age of eight years are at risk of being overlooked or neglected, as these young individuals may struggle to recognize their negative emotions due to limited emotional awareness (15). This is particularly true for vulnerable children, where issues related to self-reporting can impact the clinical assessment (16). Existing screening tools often consist of extensive parent-reported surveys, which inherently result in the under-reporting of children’s symptoms. This occurs because discerning a child’s thoughts and emotions can be challenging, even for adults who are intimately familiar with the child’s behavior (17, 18). These challenges underscore the need for an objective, accurate, and easily applicable mental health screening tool to detect anxiety and depression in young children (19).

### 1.3 Mental health screening using digital biomarkers

Reliable measurements of mental health are crucial for patient care and clinical research. As such, the use of digital technologies for assessing mental health is increasing, aiming to overcome the limitations of conventional evaluations by objectively measuring mental health biomarkers (20). Digital biomarkers refer to objective, measurable physiological and behavioral indicators collected through portable, wearable, implantable, or ingestible digital devices (21). Across various medical apparatus, hardware, or software strata, digital biomarkers can capture physiological and behavioral cues from individuals (22). The use of these digital biomarkers enables the continuous remote monitoring and evaluation of reliable clinical data, thereby enhancing the accuracy of diagnoses and treatments (23, 24). Moreover, these devices are less susceptible to human bias and can reduce patient stress (25). In fact, a growing body of research is exploring tools that use digital biomarkers for assessing various aspects of depression, anxiety, and stress (26, 27).

In clinical or laboratory settings, a wide array of digital biomarkers is available for conducting mental health screenings. However, our study focuses on developing a user-friendly, non-clinical screening tool designed for home use, especially for children. To achieve this goal, we are exploring digital biomarkers that can be developed through software and implemented on universally accessible devices.

#### 1.3.1 Heart rate variability

Heart rate variability (HRV) denotes the variation between consecutive heartbeats, facilitating optimal adaptation to external stressors. HRV is influenced by the activation of the parasympathetic autonomic system, particularly through the vagus nerve, which decelerates the heart rate, and sympathetic activation, which accelerates the heart rate (28). Commonly used time domain HRV parameters include the standard deviation of the interbeat intervals of normal sinus beats (SDNN), the root mean square of successive differences (RMSSD), and the percentage of successive RR intervals that differ by more than 50 milliseconds (pNN50). Meanwhile, frequency domain parameters consist of high frequency (HF), low frequency (LF), and their ratio (LF/HF). The RMSSD and pNN50 indicate parasympathetic influence, whereas the SDNN reflects both sympathetic and parasympathetic modulation. Additionally, LF responds to both sympathetic and parasympathetic activities, and HF primarily reflects parasympathetic actions (29).

Numerous studies have explored the relationship between HRV and conditions such as depression, anxiety, and stress. Decreased HF, LF, and RMSSD, along with an increased LF/HF ratio, are associated with depression (28, 30). This pattern is also observed in pediatric and adolescent populations. According to a recent meta-analysis (31), individuals with Major Depressive Disorder exhibit reduced HRV measures such as HF, RMSSD, and pNN50. Furthermore, reduced HF, LF, RMSSD, and SDNN, coupled with an elevated LF/HF ratio, have been linked to anxiety (32). In a study comparing HRV between healthy control groups and children with anxiety disorders, decreased LF and HF were also observed in the children with anxiety disorders (33). HR, HF, LF, LF/HF, pNN50, and RMSSD have been identified as features associated with stress (34).

#### 1.3.2 Eye-tracking

Human mental health issues, such as depression and anxiety, are associated with eye movements. A significant factor influencing the onset, persistence, and reappearance of depression is the biased attention towards emotional stimuli (35). Researchers have suggested that individuals with depression exhibit an attraction to negative stimuli and struggle to divert their attention away from such stimuli (36). Recent meta-analysis research supports this claim, indicating that those with depression tend to have an attention bias towards sad faces or melancholic images, along with a reduced attention to positive images (37). Clinically, anxious individuals, as well as those with a propensity for anxiety, show an attention bias towards threat stimuli (38). Children with anxiety disorders are more vigilant or attentive to potential dangers in their environment (39). These studies have used fixation time or reaction time following stimulus presentation to measure attention bias in depression and anxiety.

#### 1.3.3 Voice

Empirical evidence indicates that mood changes are reflected in one’s voice, with ongoing research assessing depressive and stress states using these digital biomarkers (40, 41). Recent research involving individuals aged 6-16 diagnosed with Major Depressive Disorder and Bipolar Disorder, as well as healthy controls, has observed significant differences in voice characteristics among these groups (42). Children with a predisposition for depression exhibit lower-pitch voices and more repetitive speech inflections and content compared to children in the control group (43). While research on children’s voices is limited, studies involving adults with depression have reported significant differences between clinical and control groups, such as decreased pitch (44) and increased jitter (45).

#### 1.3.4 Multimodal digital biomarkers

The integration of various digital biomarkers could be immensely beneficial for assessing mental health status. Employing digital biomarkers in a multimodal approach can be meaningful, as each set of data can complement the other (46). Context-appropriate multimodalities offer significant advantages in assessing one’s state of mind (47). Indeed, several studies have combined traditional biomarkers (e.g., salivary cortisol and sweat) with digital biomarkers (48), or integrated various digital biomarkers with each other (e.g., HRV, voice, electroencephalogram, and movement data) (49, 50). However, many studies are currently limited to using digital biomarkers available in a laboratory environment, indicating a need for practical research to establish their efficacy. Moreover, while these approaches are common in adults, research in children using HRV, eye-tracking, and voice in a multimodal approach is rare, as far as we are aware. Recent systematic reviews have discussed the potential for passive phenotypes in children and adolescents (51), which could serve as an objective measurement method. Nonetheless, there is a possibility that children may not have access to their own smartphones or wearable devices. Thus, it is essential to collect digital biomarker data through tasks. The evaluation of children’s mental health using movement and voice targeted children aged 4-8 years, with the study by Loftness et al. (19) being a notable exception. In that study, active phenotype data were obtained by using tasks that children can perform, similar to our study. To bridge this research gap and explore the potential for both research and clinical utility, we have developed an application that can be used across various settings.

### 1.4 Research objective and hypotheses

Our primary objective was to confirm the feasibility of using various digital biomarkers in children, specifically for monitoring childhood depression, anxiety, and stress. Based on previous research, the following hypotheses were formulated:

H1. HRV, eye-tracking, and voice features will account for the child’s depression score.

H2. HRV, eye-tracking, and voice features will account for the child’s anxiety score.

H3. HRV, eye-tracking, and voice features will account for the child’s stress score.

## 2 Materials and methods

### 2.1 Feature exploration

We initially examined previous studies that assessed depression, anxiety, and stress using HRV, eye-tracking, and voice data. Based on these references, we selected variables with a significance level of < 0.005 to avoid multicollinearity. The variables commonly used, as cited in previous academic papers, are summarized in **Table 1**.

**Table 1:** Digital biomarker variables used in analyses.

Digital Biomarkers	Features	Description
Heart Rate Variability	HF	Absolute power of the high-frequency band (0.15–0.4 Hz)
	LF	Absolute power of the low-frequency band (0.04–0.15 Hz)
	LF/HF	Ratio of LF-to-HF power
	RMSSD	Square root of the mean squared differences of successive heart periods
	SDNN	Standard deviation of NN intervals
	pNN50	Percentage of successive RR intervals that differ by more than 50milliseconds
Eye-tracking	Total Fixation Time	Total duration of gaze on the stimuli
Voice	Pitch	Relative highness or lowness of a tone as perceived by the ear, which depends on the number of vibrations per second produced by the vocal cords
	Jitter	Variation of the phase of a timing signal from its ideal positions in time
	Shimmer	Measure of the high-frequency amplitude perturbations in a speech signal
	Loudness	Subjective perception of the intensity or volume of a sound as perceived by the human auditory system

### 2.2 Digital assessment tool

**Figure 1:**
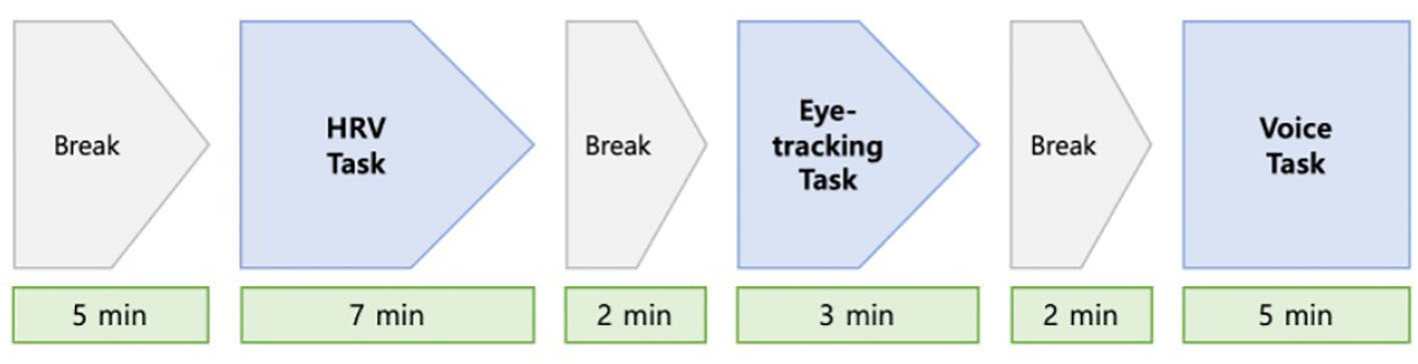
Measurement process for subject assessment.

The application we developed performs a comprehensive evaluation by simultaneously incorporating three standard tests: HRV, eye-tracking, and voice (**Figure 1**).

#### 2.2.1 Heart rate variability

The HRV task employed a remote photoplethysmography (rPPG) method with a camera sensor to collect HRV data. Remote photoplethysmography allows for non-contact screening of cardiovascular activity by detecting pulse-induced subtle color changes on the human skin surface using a multi-wavelength RGB camera. In our study, a tablet personal computer (PC) equipped with a biomarker collection module application was used for this purpose.

Specific conditions were established during data collection to ensure the data’s suitability for analysis. First, a minimum of three minutes of uninterrupted data was required. Second, the participants’ faces had to be fully visible, with appropriate illumination and minimal noise being essential. Third, a stable state was established beforehand to facilitate the accurate measurement of heart rate variability.

**Figure 2:**
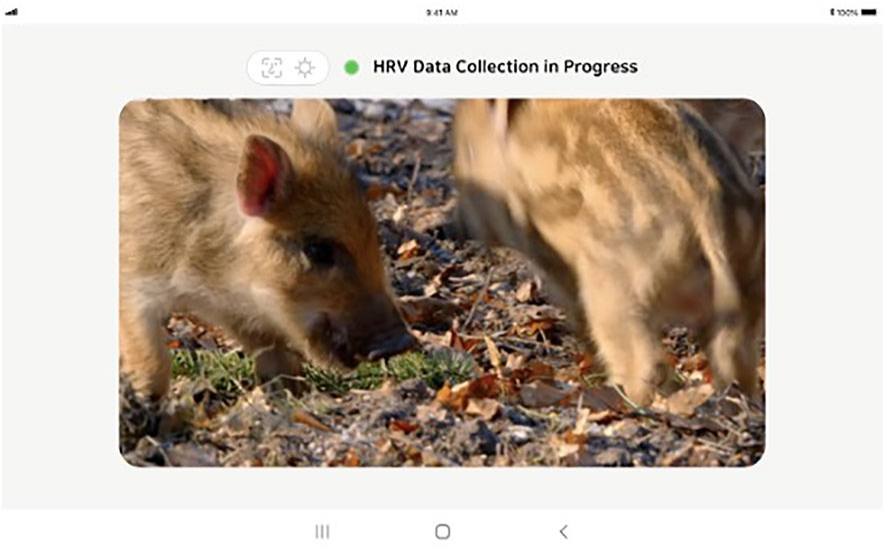
Tablet screen used for measuring heart rate variability.

Before the examination, the height of the tablet holder was adjusted to align with the participant’s face, with the tablet positioned horizontally. Considering the challenge children face in maintaining stillness for the designated HRV data collection period, we used nature videos during the HRV measurements, following the approach of prior studies focused on children (52, 53). A pre-guided video featuring natural scenery was then played for calibration, lasting approximately 200 seconds. After completing the calibration, HRV data were collected for 180 seconds, with careful monitoring for any factors that might interfere with data collection (**Figure 2**). If any difficulty in data collection was signaled by a phrase accompanied by a red circle during the test, the researcher would assess the child’s posture and instruct adjustments to the tablet holder for improved data collection, focusing on face recognition, lighting, and noise.

We employed two widely accepted methodologies for extracting HRV variables: time-domain and frequency-domain analyses. The HRV data, sampled at a rate of 30 Hz, generated outputs approximately every two seconds. In the frequency domain, we used the Fast Fourier Transform (FFT) to decompose the pulse wave signal into Low-Frequency (LF) and High-Frequency (HF) domains. Various parameters, such as pNN20, pNN50, SDNN, and RMSSD, were computed using a sliding window approach with a window size of 30 Inter-Beat Intervals (IBI) datapoints. This approach means that these indices were recalculated each time a new IBI was obtained.

#### 2.2.2 Eye-tracking

The eye-tracking task employed a software development kit (SDK) dedicated to eye-tracking, which collects eye-tracking markers using the front camera sensor. Initially, the SDK establishes a baseline for the participant’s gaze coordinates through calibration. This calibration involves the participant following a moving red dot across each vertex, typically taking approximately 3-5 seconds to complete. Subsequently, the SDK captures data on the participant’s gaze position based on these baseline coordinates.

The attentional bias task used in the eye-tracking measurement is a widely recognized research tool. It involves analyzing the movement of the participant’s gaze while they are exposed to photographs of emotional facial expressions, such as happiness, sadness, contempt, or neutral. This task identifies the participant’s tendency to selectively focus on specific emotional stimuli, providing valuable insights into emotional processing and attention allocation across various contexts.

**Figure 3:**
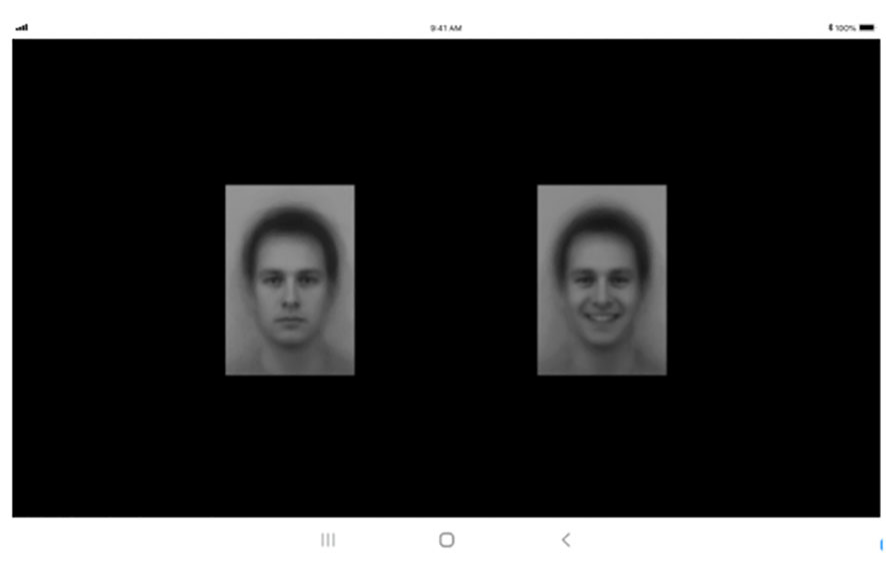
Tablet screen used for measuring eye-tracking movements.

**Figure 4:**
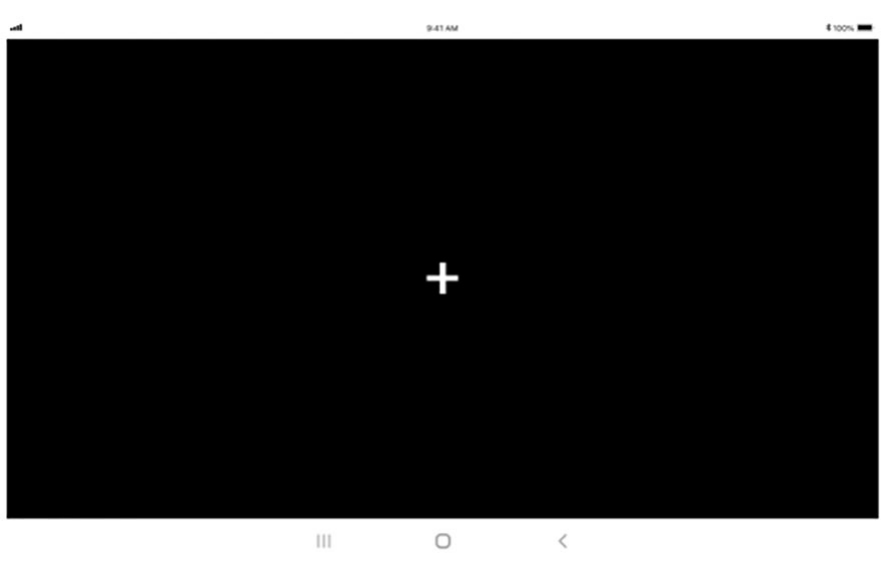
Tablet screen coordinates for preventing fixation.

Before starting data collection, the researcher adjusted the distance between the participant’s eyes and the examination tool to 60 centimeters. The children were asked if they could clearly see the tablet screen. They were informed that a “+” mark and a face photo would appear alternately on the screen, and were instructed to freely observe the face photo when it appeared while focusing on the ‘+’ mark. After calibration, an emotional face stimulus was displayed for three seconds, repeating this process 36 times (**Figure 3**). The execution of this task followed methodologies used in prior studies (54), which helps prevent non-compliance issues such as excessive movement, inability to respond to the ‘+’ mark, or gaze avoidance (**Figure 4**). The researcher monitored to ensure that participants maintained their focus on the tablet screen throughout the task.

#### 2.2.3 Voice

**Figure 5:**
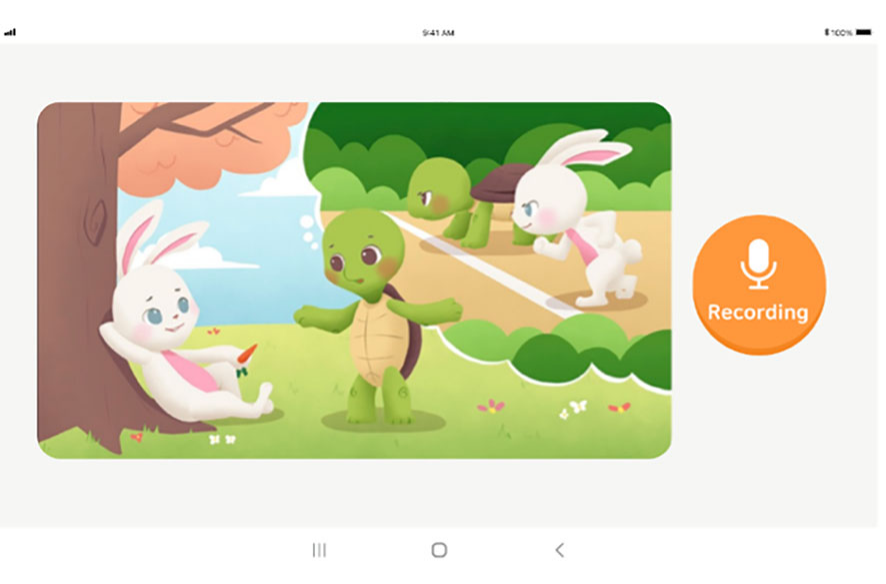
Tablet screen for performing the task of “speaking about image”.

**Figure 6:**
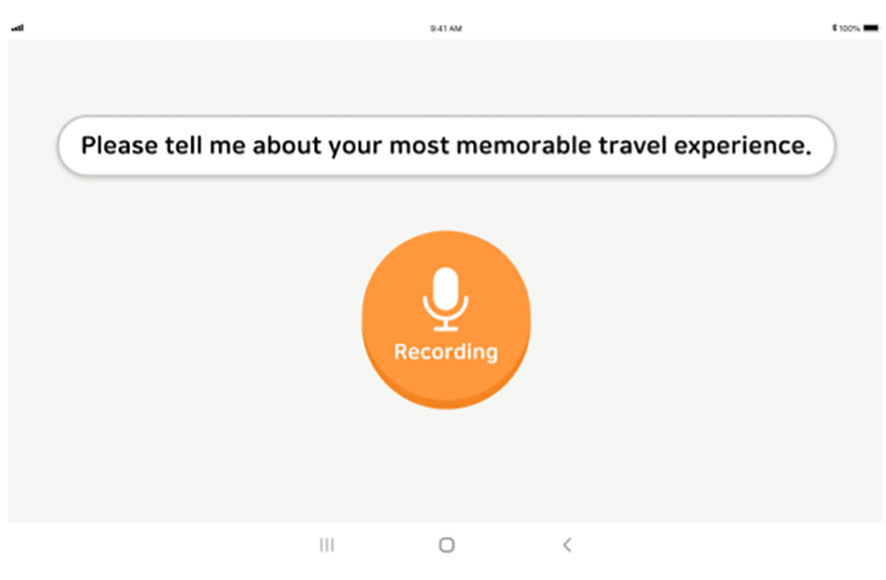
Tablet screen for performing the task of “speaking about subject”.

Voice data for biomarker collection were obtained through two distinct tasks. The first task involved presenting image stimuli to the subjects and recording their voices as they described the images’ content, referred to as “speaking about image” (**Figure 5**). In the second task, subjects were prompted to freely discuss a specific topic, referred to as “speaking about subject” (**Figure 6**). The primary aim of both tasks was to analyze the subjects’ voice features under a general free speech scenario. This method sought to capture the inherent characteristics of the subjects’ voices in natural and unstructured speech contexts. All initial voice data were recorded using the tablet’s recording feature and stored locally before being transmitted to the server.

The task “speaking about image” commenced with participants viewing a series of illustrations on the display. Subjects were encouraged to freely and voluntarily express their interpretations of the scenes while the researcher recorded the process. If a participant’s speaking time exceeded two minutes, the researcher proceeded to the next task. For the “speaking about subject” task, participants were given a specific topic, such as “travel”, and asked to share their experiences related to the topic within a two-minute timeframe. All participants’ utterances were recorded. The data collection process concluded after both the picture speech and the subject speech tasks were completed. In instances where the volume of voice data was deemed insufficient, the researcher employed a specific protocol to elicit more speech from the participants, involving posing questions that prompted further utterance. An example of a speech induction question was: “(While showing pictures of travel destinations) Where do you want to go the most? Why?” This task adopted the methods used in previous studies to assess depression and anxiety (55).

We used the Praat package (56), a tool common in previous voice research, to extract features from the voice recording data. Praat is widely acknowledged as a leading toolbox for voice data feature extraction (57) and serves as an ideal platform for developing a prosodic feature extraction tool in the public domain, enabling further use and expansion by other researchers (58).

To label the collected data for each participant, basic demographic information was recorded at the initial access to the biomarker collection module application. The researcher entered the participant’s name, gender, and date of birth on the input screen and then pressed the confirmation button, enabling us to accurately associate the collected data with each individual participant for further analysis and interpretation.

### 2.3 Participants and procedure

We conducted the study procedures in the clinical laboratory of Chung-Ang University Medical School. Participants were recruited voluntarily after they and their parents expressed interest through online postings and promotional materials, leading to the enrollment of 54 children. Given the focus on elementary school students, the age range of participants was seven to 11 years. There were no dropouts, and written consent was obtained from all participants. Upon their arrival at the laboratory, parents and/or guardians and children were guided through the consent procedures by researchers from Chung-Ang University. After giving consent, participants filled out demographic questionnaires and trait inventories, underwent a dominant hand test, and then used the digital assessment tool. Upon completion, each participant was informed that the test had concluded and they were reunited with their parents. We adhered to a heuristic of 50 people per condition, based on sample sizes from previous literature (59). Ethical approval for the study was obtained from the Institutional Review Board at Chung-Ang University.

The biomarker collection module, developed as a digital application compatible with the Android operating system, was designed for future real-world application. HRV and eye-tracking data collection was facilitated by Software Development Kits (SDKs), while voice data were captured using the tablet’s built-in recording function. All collected data were initially stored locally on the devices and later transmitted to our server upon completion of the process. For biomarker extraction, we employed Samsung tablet PCs, which facilitated efficient and accurate data collection in a controlled environment.

Additionally, participants were given a 2-minute break before each task in our assessment tool to ensure a stable state.

### 2.4 Measures

#### 2.4.1 Biophysiological measurements

Three digital biomarkers—HRV, voice, and eye-tracking—were collected from children using the application we developed. In our digital biomarker measurement protocol, we employed custom-developed tools to assess HRV, eye-tracking, and voice. Participants were advised to avoid activities that could potentially influence their physiological data on the day of testing, such as engaging in vigorous exercise or consuming caffeine-containing carbonated beverages one hour before the assessment. The assessments lasted approximately 40 minutes and were conducted in quiet environments with controlled temperatures ranging from 22°C to 25°C. A researcher was present alongside the participants during the testing.

#### 2.4.2 Clinical measures

We collected data from participants through self-reported measures. Children completed self-assessment measures for depression, anxiety, and stress.

Depressive symptoms in children were assessed using the Children’s Depression Inventory, 2nd edition (CDI-2), which consists of 28 items divided into two primary factors: emotional problems and functional problems. Children rated these items on a 3-point Likert scale from 0 (no symptoms) to 2 (clear symptoms), with higher scores on the CDI-2 subscales indicating more depressive symptoms (60). The validated Korean version, K-CDI-2, was used in our assessments, demonstrating good internal consistency with α = .86, comparable to the original version’s α = .91 (61). The internal consistency reported in our study was α = .869, and T scores were used for measurement.

Anxiety in children was evaluated using the Revised Children’s Manifest Anxiety Scale, 2nd edition (RCMAS-2), which includes 49 items across five scales: physiological anxiety, defensiveness, worry, inconsistent responding, and social anxiety. It is a self-report instrument designed to assess chronic and significant anxiety in children (62). The validated K-RCMAS-2 was used (63), with our study reporting an internal consistency of α = .991. Total scores were used for measurement.

The stress measure in this study was based on a daily life scale developed by Kim (64) for elementary school students, using a subset of 10 items from the original 65-item scale by selecting the two items with the highest correlation values among the five factors. This approach was chosen anticipating the challenges children might face in self-reporting on all 65 items. The internal consistency reported in our study was α = .818, with total scores used for measurement.

### 2.5 Statistical analyses

Data underwent partial correlation analyses, accounting for age and gender influences, followed by hierarchical regression analysis to determine the impact of digital biomarker features on mental health symptoms. In these analyses, age and gender were accounted for as covariates in the first block. Digital biomarker features were then introduced in a stepwise multiple regression in the second block. This covariate analysis was conducted to address the broad age range and potential gender influence on digital biomarker data. Accordingly, partial correlation analyses were carried out, considering the effects of age and gender on the observed associations. This analytical approach was used in a study investigating the relationship between multimodal digital biomarker features and major depressive disorder scores (65).

Furthermore, the sample data were categorized by the children’s age into two groups: elementary lower grades (1st, 2nd, 3rd grades) and elementary upper grades (4th, 5th, 6th grades). This categorization enabled the exploration of potential age-related influences on digital biomarker data. Given the hypothesis that the broad age range might impact digital biomarker outcomes, participants were divided into two groups for subsequent correlation analysis. The statistical significance level was established at α = 0.05.

## 3 Results

### 3.1 Sample characteristics

The final sample consisted of 43 participants. Six participants were excluded due to missing HRV data. This exclusion was necessary to ensure a complete dataset for analyzing the potential of using digital biomarkers in a multimodal context. Outliers were identified and removed when five or more data points deviated more than three standard deviations (SDs) from the mean of each digital biomarker variable. Ultimately, the sample included 25 males and 18 females, with an average age of 9.05 years (SD = 1.308) (**Table 2**).

**Table 2:** Sample characteristics.

	Participants (n = 43)				
	Min	Max	Mean	Median	Standard Deviation
Sex ratio (m/f)	25/18				
Age (years; mean ± SD)	7	11	9.05	9	± 1.308
Intelligence quotient (IQ)	70	139	107.372	110	± 15.922
K-CDI-2 (T-value)	40	70	50.79	49	± 9.729
K-RCMAS-2	0	64	12.67	9	± 11.647
Stress scale	13	34xw	20.88	20	± 5.556

Min, minimum; Max, maximum; m, male; f, female; SD, standard deviation; K-CDI-2, Korean Children’s Depression Inventory, 2nd edition; K-RCMAS-2, Korean Revised Children’s Manifest Anxiety Scale, 2nd edition.

### 3.2 Statistical correlations

To explore the relationships among children’s depressive symptoms, anxiety symptoms, and stress with digital biomarker variables, we conducted partial correlation analyses. This method enabled the identification of pure correlations by controlling for age and gender effects. No significant correlations were found between HRV variables and age or gender. However, significant associations were observed between eye-tracking variables and gender, with females spending more time viewing contemptuous and sad expressions. Additionally, we have detailed the interrelations among the digital biomarkers in **Table 3**.

**Table 3:** Correlations among digital biomarker features.

	HR	HF	LF	LF/HF	RMSSD	SDNN	pNN50	Happy. TF:time	Neutral. TF:time	Sad. TF:time	Contempt.TF:time	Pitch_ mean	Jitter	Shimmer	Loudness_mean
HR	**-**														
HF	-0.090														
LF	-0.245	-.442**													
LF/HF	-0.004	-.794***	.765***												
RMSSD	-.410**	.355*	0.174	-0.148											
SDNN	-.668***	.371*	0.128	-0.229	.752***										
pNN50	-.390**	0.189	0.284	-0.055	.797***	.547***									
Happy. TF:time	-0.034	-0.116	-0.117	-0.053	-0.022	0.074	-0.052								
Neutral. TF:time	-0.025	-0.105	0.025	0.024	0.084	0.034	0.078	.620***							
Sad. TF:time	0.037	0.042	-0.158	-0.094	-0.011	-0.126	0.044	.471**	.687***						
Contempt.TF:time	-0.123	0.167	-0.101	-0.121	0.037	0.082	-0.002	.343*	.354*	.539***					
Pitch _mean	0.069	-0.088	0.223	0.131	0.063	-0.109	0.090	0.128	0.111	0.046	0.080				
Jitter	-0.221	-0.289	.345*	.358*	0.124	0.182	0.120	0.091	0.278	-0.041	0.059	0.147			
Shimmer	-0.107	-0.073	0.186	0.206	0.173	0.040	0.110	-0.004	0.202	-0.008	0.005	-0.035	.694***		
Loudness_mean	0.204	0.240	-0.203	-0.188	-0.027	-0.116	-0.115	-0.018	-0.250	-0.063	0.063	-0.095	-.706***	-.680***	–

HR, Heart Rate; HF, High-Frequency Power; LF, Low-Frequency Power; LF/HF, Ratio of Low-Frequency to High-Frequency; RMSSD, Root Mean Square of the Successive Difference; SDNN, Standard Deviation of Normal to Normal Interval; pNN50, Proportion of NN50 divided by the total number of NN (R-R) intervals; Happy.TF,time, The total duration of gazing at happy facial expressions; Neutral.TF,time, The total duration of gazing at neutral facial expressions; Sad.TF,time, The total duration of gazing at sad facial expressions; Contempt.TF,time, The total duration of gazing at contemptuous facial expressions.

*p <.05, **p <.01 ***p <.001.

The K-CDI-2 and K-RCMAS-2 scores showed correlations with HRV variables. The K-CDI-2 was positively correlated with LF (r = 0.409, *p* = 0.008) (**Table 4**) and negatively associated with eye-tracking variables (r = -0.374, *p* = 0.016) (**Table 4**). Conversely, the K-RCMAS-2 displayed a negative correlation with HR (r = -0.310, *p* = 0.048), HF (r = -0.340, *p* = 0.030), and a positive relationship with LF/HF (r = 0.409, *p* = 0.008) (**Table 5**). For the stress scale, pNN50 showed a negative correlation (r = -0.382, *p* = 0.014). Acoustic variables (voice) were not correlated with the clinical scales (**Table 6**).

**Table 4:** Partial correlations among HRV and clinical measurements.

	HR	HF	LF	LF/HF	RMSSD	SDNN	pNN50
K-CDI-2	-0.260	-0.050	0.409**	0.303	0.054	0.169	-0.055
K-RCMAS-2	-0.310*	-0.340*	0.295	0.409**	-0.135	0.070	-0.220
Stress	-0.124	-0.133	0.118	0.275	-0.300	0.024	-0.382*

Controlling for gender and age as covariates. K-CDI-2, Korean Version Children Depression Inventory 2^nd^ Edition; K-RCMAS-2, The Revised Children’s Manifest Anxiety Scale Korean Version 2^nd^ Edition; Stress, Stress Scale; HR, Heart Rate; HF, High-Frequency Power; LF, Low-Frequency Power; LF/HF, Ratio of Low-Frequency to High-Frequency; RMSSD, Root Mean Square of the Successive Difference; SDNN, Standard Deviation of Normal to Normal Interval; pNN50, The proportion of NN50 divided by the total number of NN (R-R) intervals.

*p <.05, **p <.01.

**Table 5:** Partial correlations among eye-tracking and clinical measurements.

	Happy.TF:time	Neutral.TF:time	Sad.TF:time	Contempt.TF:time
K-CDI-2	-0.185	-0.171	-0.374**	-0.181
K-RCMAS-2	0.044	-0.108	-0.164	-0.059
Stress	0.174	0.001	-0.099	0.164

Controlling for gender and age as covariates. K-CDI-2, Korean Version Children Depression Inventory 2nd Edition; K-RCMAS-2, The Revised Children’s Manifest Anxiety Scale Korean Version 2nd Edition; Stress, Stress Scale; Happy.TF:time, The total duration of gazing at happy facial expressions; Neutral.TF:time, The total duration of gazing at neutral facial expressions; Sad.TF:time, The total duration of gazing at sad facial expressions; Contempt.TF:time, The total duration of gazing at contemptuous facial expressions.

**p <.01.

**Table 6:** Partial correlations among voice and clinical measurements.

	Pitch_mean	Jitter	Shimmer	Loudness_mean
K-CDI-2	-0.185	0.181	0.030	-0.152
K-RCMAS-2	-0.006	0.200	-0.019	-0.188
Stress	0.002	0.288	0.007	-0.130

Controlling for gender and age as covariates. K-CDI-2, Korean Version Children Depression Inventory 2nd Edition; K-RCMAS-2, The Revised Children’s Manifest Anxiety Scale Korean Version 2nd Edition; Stress, Stress Scale.

Further analyses were conducted to examine the impact of participants’ varied ages. Anticipating that the broad age range could influence digital biomarker outcomes, participants were stratified into two groups for subsequent correlation analysis: lower grades (grades 1 to 3) and upper grades (grades 4 to 6) of elementary school, with gender controlled in our analyses (**Tables 7**, **8**).

**Table 7:** Partial correlations among digital biomarker features in lower grades.

	HR	HF	LF	LF/HF	RMSSD	SDNN	pNN50	Happy. TF:time	Neutral. TF:time	Sad. TF:tim-.061e	Contempt.TF:time	Pitch_ mean	Jitter	Shimmer	Loudness_mean
K-CDI-2	-.328	.503*	.237	-.273	.399	.428	.415	-.355	-.239	-.503*	-.151	.002	-.081	-.060	-.073
K-RCMAS-2	-.369	-.013	-.149	-.087	.002	.248	-.112	-.142	-.017	-.061	.061	-.261	-.031	.011	-.121
Stress	-.060	.348	-.280	-.341	-.133	.055	-.123	-.084	-.013	-.227	.001	-.126	.464*	.295	-.124

HR, Heart Rate; HF, High-Frequency Power; LF, Low-Frequency Power; LF/HF, Ratio of Low-Frequency to High-Frequency; RMSSD, Root Mean Square of the Successive Difference; SDNN, Standard Deviation of Normal to normal Interval; pNN50, The proportion of NN50 divided by the total number of NN (R-R) intervals; Happy.TF:time, The total duration of gazing at happy facial expressions; Neutral.TF:time, The total duration of gazing at neutral facial expressions; Sad.TF:time, The total duration of gazing at sad facial expressions; Contempt.TF:time, The total duration of gazing at contemptuous facial expressions.

*p <.05.

**Table 8:** Partial correlations among digital biomarker features in upper grades.

	HR	HF	LF	LF/HF	RMSSD	SDNN	pNN50	Happy. TF:time	Neutral. TF:time	Sad. TF:time	Contempt.TF:time	Pitch_ mean	Jitter	Shimmer	Loudness_mean
K-CDI-2	-.160	-.351	.499*	.499**	-.159	-.025	-.291	-.008	-.070	-.157	-.096	-.082	.396	.126	-.235
K-RCMAS-2	-.294	-.431*	.443*	.529*	-.203	-.018	-.262	.141	-.143	-.203	-.027	.091	.314	-.020	-.229
Stress	-.141	-.326	.319	.489*	-.4118	-.027	-.523*	.342	.006	-.017	.274	-.009	.235	-.157	-.113

Controlling for gender as covariates. K-CDI-2: Korean Version Children Depression Inventory 2^nd^ Edition; K-RCMAS-2, The Revised Children’s Manifest Anxiety Scale Korean Version 2^nd^ Edition; Stress, Stress Scale; HR, Heart Rate; HF, High-Frequency Power; LF, Low-Frequency Power; LF/HF, Ratio of Low-Frequency to High-Frequency; RMSSD, Root Mean Square of the Successive Difference; SDNN, Standard Deviation of Normal to Normal Interval; pNN50, The proportion of NN50 divided by the total number of NN (R-R) intervals; Happy.TF:time, The total duration of gazing at happy facial expressions; Neutral.TF:time, The total duration of gazing at neutral facial expressions; Sad.TF:time, The total duration of gazing at sad facial expressions; Contempt.TF:time, The total duration of gazing at contemptuous facial expressions.

*p <.05, **p <.01.

The HF value, not significantly associated with depression scores, showed a significant positive correlation in the lower-grade group (r = 0.503, *p* = 0.028). Similarly, the LF value, not significantly correlated with anxiety scores, demonstrated a significant positive relationship in the upper-grade group (r = 0.489, *p* = 0.016). Conversely, jitter was positively associated (r = 0.464, *p* = 0.046) with stress in the low-grade group. These results reveal relationships for HRV and voice features that were previously not correlated.

### 3.3 Statistical regressions

We assessed the predictive capabilities of digital biomarker variables for depressive symptoms, controlling for age and gender as covariates. Initially, age and gender were included as covariates, and subsequently, HF, LF, Sad.TF:time(refers to the total duration of gazing at sad facial expressions), pitch, jitter, and shimmer were introduced stepwise. This approach was adopted because these variables have been identified as predictors of depression in prior research. LF and Sad.TF:time emerged as significant predictors for the total depressive symptom score, yielding the following results: F(4, 38) = 4.103, *p* = 0.007, R² = 0.302, and ΔR² = 0.228 (**Table 9**).

**Table 9:** Regression analysis prediction of a depressive symptom composite from digital biomarker variables, with covariance for age and gender.

		B	Std. Error	β	*t*	Sig.	R	ΔR²
Model 1	(Constant)	50.803	10.803		4.703	<.001	0.231	0.006
	Age	-0.205	1.155	0.225	1.451	0.155		
	Gender	4.394	3.028		1.867	0.069		
Model 2 Step 1	(Constant)	25.252	13.525		1.867	0.069	0.460	0.151
	Age	-0.113	1.068	-0.015	-0.106	0.916		
	Gender	4.352	2.798	0.223	1.555	0.128		
Step 2	LF	0.199	0.071	0.398	2.800	0.008		
Model 3 Step 1	(Constant)	29.031	13.010		2.231	0.32	0.549	0.228
	Age	0.697	1.082	0.094	0.644	0.523		
	Gender	6.909	2.908	0.354	2.376	0.023		
Step 2	LF	0.167	0.069	0.346	2.515	0.016		
	Sad.TF:time	-0.987	0.449	-0.343	-2.211	0.033		

LF, Low- Frequency Power; Sad.TF:time, The total duration of gazing at sad facial expressions.

We performed the same analysis for anxiety symptoms, following a similar procedure. This time, variables associated with anxiety symptoms in the literature, such as HF, LF, LF/HF, HR, pNN50, Contempt.TF:time(refers to the total duration of gazing at contemptuous facial expressions), pitch, jitter, and shimmer, were included in the second block. LF/HF, HR, and pNN50 remained significant predictors for the total anxiety symptom score, with results as follows: F(5, 37) = 5.565, *p* < 0.001, R² = 0.429, and ΔR² = 0.352 (**Table 10**).

**Table 10:** Regression analysis prediction of an anxiety symptom composite from digital biomarker variables, with covariance for age and gender.

		B	Std. Error	β	*t*	Sig.	R	ΔR²
Model 1	(Constant)	1.024	12.814		0.080	0.937	0.266	0.024
	Age	1.010	1.370	0.114	.737	0.465		
	Gender	5.995	3.592	.257	1.669	0.103		
Model 2 Step 1	(Constant)	-9.081	12.378		-.734	.468	0.476	0.167
	Age	.755	1.269	.085	.595	.556		
	Gender	5.550	3.323	.238	1.670	.103		
Step 2	LF/HF	14.137	5.047	.396	2.801	.008		
Model 3 Step 1	(Constant)	226.594	106.463		2.128	.040	0.562	0.244
	Age	.855	1.210	.096	.707	.484		
	Gender	4.571	3.196	.196	1.430	.161		
Step 2	LF/HF	14.127	4.809	.396	2.938	.006		
	HR	-3.001	1.347	-.302	-2.227	.032		
Model 4 Step 1	(Constant)	362.806	110.595		3.280	.002	0.655	0.352
	Age	1.068	1.123	.120	.951	.348		
	Gender	4.010	2.966	.172	1.352	.185		
Step 2	LF/HF	13.367	4.460	.374	2.997	.005		
	HR	-4.475	1.360	-.451	-3.290	.002		
	pNN50	-2.112	.778	-.369	-2.713	.010		

HR, Heart Rate; LF/HF, Ratio of Low-Frequency to High-Frequency; pNN50, The proportion of NN50 divided by the total number of NN (R-R) intervals.

For stress symptoms, we analyzed variables linked to stress in previous studies (HF, pNN50, Contempt.TF:time, pitch, jitter, and shimmer) in the second block. Here, pNN50 stood out as a significant predictor for the total stress symptom score: F(3, 39) = 2.545, *p* = 0.03, R² = 0.164, and ΔR² = 0.099 (**Table 11**).

**Table 11:** Regression analysis prediction of a stress symptom composite from digital biomarker variables, with covariance for age and gender.

		B	Std. Error	β	*t*	Sig.	R	ΔR²
Model 1	(Constant)	34.266	10.237		3.347	0.002	0.235	0.008
	Age	1.628	1.096	0.232	1.486	0.145		
	Sex	1.758	2.793	0.098	0.629	0.533		
Model 2 Step 1	(Constant)	48.686	11.670		4.172	<.001	0.405	0.099
	Age	1.653	1.044	0.236	1.583	0.121		
	Sex	1.609	2.662	0.090	0.604	0.549		
Step 2	pNN50	-1.453	.646	-0.330	-2.250	0.03		

pNN50, The proportion of NN50 divided by the total number of NN (R-R) intervals.

## 4 Discussion

The primary objectives of our study were to assess the possibility and feasibility of monitoring depression, anxiety, and stress in children through the collection of digital biomarkers. Our analysis revealed three significant findings, offering valuable insights for future clinical applications.

### 4.1 Application of the multimodal approach to depression

Our research suggested the potential clinical utility of multimodal digital biomarkers in tracking childhood depression. While the effect size was modest, the total duration spent viewing sad faces was identified as an explanatory variable for child depression, particularly in relation to LF. These outcomes support our first hypothesis and underscore the potential of multimodal biomarkers in monitoring depression among children, marking a novel application of this method in pediatric populations. This discovery paves the way for further exploration of childhood depression using a multimodal approach in future studies.

### 4.2 Explanatory variables for anxiety

Moreover, anxiety symptoms were most accurately explained by HRV features. Notably, HRV was one of the variables that explained both depression and stress. However, the variables explaining anxiety solely consisted of HRV features, which exhibited the greatest explanatory power in our findings. We propose that the effectiveness of HRV stems from its ability to capture variations in autonomic nervous system (ANS) activity. Prior studies have consistently demonstrated a significant relationship between ANS activity and depression, anxiety, and stress (66), lending support to our hypothesis.

### 4.3 Age differences in correlation analysis

Finally, we conducted additional analyses based on participants’ ages, dividing them into lower grades (grades 1 to 3) and upper grades (grades 4 to 6) of elementary school. Our findings align with previous research that investigated age-related variations in digital biomarker patterns (67, 68). Importantly, our research identified distinct digital biomarker patterns in children across different age groups, including both before and during adolescence. This finding highlights the presence of age-specific disparities in digital biomarker patterns.

### 4.4 Study limitations

Our study, while producing potentially beneficial results, was subject to several limitations.

First, our small sample was characterized by a broad age range. The diverse developmental stages of the children, corresponding to their ages, may have influenced our results. A sample size calculation based on a large effect size of 0.4 indicated the need for 49 participants, highlighting the insufficiency of our initial sample. However, the effect size derived from our findings was 0.6, suggesting the sample size was adequate. However, to enhance the credibility of our findings, recruiting children within a narrower age range and increasing the sample size are essential for more definitive conclusions. This limitation reflects the exploratory nature of our study and indicates the need for further research.

Second, our study did not include children clinically diagnosed with depression or anxiety, only healthy controls. While many previous studies have focused on diagnosed subjects, our research identified trends using clinical measurements in healthy children and relied on self-reported data rather than evaluations by caregivers or clinicians. Future studies will need to validate our assessment tool with clinically confirmed patient groups.

Third, we could not replicate prior research (68) indicating a significant decrease in LF in depressed children. However, our findings are consistent with those of Wang et al. (69), and the study by Jangpangi et al. (70), where LF was increased in the depressed group, supports our research. Further investigation is required to clarify these varied results.

As a result, our study suggests the feasibility of using multimodal biomarkers for monitoring children’s mental health in non-clinical settings but does not assert that our tool and results are suitable for clinical diagnosis.

## 5 Conclusions

Our study embarked on exploratory research to evaluate the feasibility of using a multimodal approach to understand children’s depression, anxiety, and stress. For childhood depression, LF and Sad.TF:time were identified as significant variables; for anxiety, LF/HF, HR, and pNN50; and for stress, pNN50 was the key variable. This research underscores the potential utility of multimodal digital biomarkers in children’s mental health screening, laying the groundwork for future studies. However, the limitations due to the small sample size and the exclusion of children diagnosed with depression or anxiety must be acknowledged. Our study is foundational, suggesting the need for further research to extend these findings to clinical settings. The application developed for this study leverages digital biomarkers from widely available devices, offering adaptability for non-clinical environments and paving the way for practical applications in subsequent research.

## Data availability statement

The raw data supporting the conclusions of this article will be made available by the authors, without undue reservation.

## Ethics statement

The studies involving humans were approved by The Chung-Ang University Hospital Institutional Review Board. The studies were conducted in accordance with the local legislation and institutional requirements. Written informed consent for participation in this study was provided by the participants’ legal guardians/next of kin. Written informed consent was obtained from the minor(s)’ legal guardian/next of kin for the publication of any potentially identifiable images or data included in this article.

## Author contributions

MyC: Writing – review & editing, Writing – original draft, Visualization, Software, Methodology, Formal analysis, Conceptualization. DP: Writing – review & editing, Writing – original draft, Visualization, Software, Methodology, Formal analysis, Conceptualization. MiC: Writing – review & editing, Writing – original draft, Visualization, Software, Methodology, Formal analysis, Conceptualization. SB: Writing – review & editing, Writing – original draft, Investigation. JK: Writing – review & editing, Writing – original draft, Supervision, Project administration, Funding acquisition. DH: Writing – review & editing, Writing – original draft, Supervision, Project administration, Funding acquisition.
